# Role of membrane vesicles in the transmission of vancomycin resistance in *Enterococcus faecium*

**DOI:** 10.1038/s41598-024-52310-1

**Published:** 2024-01-22

**Authors:** Johanna Lehmkuhl, Julia Sophie Schneider, Kari Lavinia vom Werth, Natalie Scherff, Alexander Mellmann, Stefanie Kampmeier

**Affiliations:** 1 Institute of Hygiene, University Hospital Münster, 48149 Münster, Germany; 2 Institute for Hygiene and Microbiology, University of Würzburg, 97080 Würzburg, Germany

**Keywords:** Bacterial pathogenesis, Infectious-disease epidemiology

## Abstract

Clonal transmission and horizontal gene transfer (HGT) contribute to the spread of vancomycin-resistant enterococci (VRE) in global healthcare. Our study investigated vesiduction, a HGT mechanism via membrane vesicles (MVs), for *vanA* and *vanB* genes that determine vancomycin resistance. We isolated MVs for VRE of different sequence types (STs) and analysed them by nanoparticle tracking analysis. Selected MV samples were subjected to DNA sequence analysis. In resistance transfer experiments, vancomycin-susceptible enterococci were exposed to MVs and bacterial supernatants of VRE. Compared to bacteria grown in lysogeny broth (MVs/LB), cultivation under vancomycin stress (MVs/VAN) resulted in increased particle concentrations of up to 139-fold (ST80). As a key finding, we could show that VRE isolates of ST80 and ST117 produced remarkably more vesicles at subinhibitory antibiotic concentrations (approx. 9.2 × 10^11^ particles/ml for ST80 and 2.4 × 10^11^ particles/ml for ST117) than enterococci of other STs (range between 1.8 × 10^10^ and 5.3 × 10^10^ particles/ml). In those MV samples, the respective resistance genes *vanA* and *vanB* were completely verifiable using sequence analysis. Nevertheless, no vancomycin resistance transfer via MVs to vancomycin-susceptible *Enterococcus faecium* was phenotypically detectable. However, our results outline the potential of future research on ST-specific MV properties, promising new insights into VRE mechanisms.

## Introduction

A major increase in deaths related to antimicrobial resistance is predicted worldwide in the upcoming years^1^. Hence, adequate treatment of bacterial infections poses a challenge to global healthcare^2^. In this context, the World Health Organisation published a list of bacteria for which research and development of new antibiotics are urgently needed. Here, the list of ‘high-priority pathogens’, containing increasingly drug-resistant bacteria, included vancomycin-resistant *Enterococcus faecium* (*E. faecium*) (VRE)^3^.

Enterococci are Gram-positive bacteria that colonise the human intestine. They are also frequently found in the environment and can survive hostile growth conditions^4^. In addition, enterococci, in particular *E.* *faecalis* and *E. faecium*, are common causes of infections in the clinical setting comprising endocarditis, bloodstream, catheter-associated and surgical site infections. Moreover, VRE bacteraemia tends to have a worse outcome than that caused by vancomycin-susceptible enterococci (VSE)^5^. Current data show rising trends towards VRE in Europe^6^. In Germany, this led to an increase in the national burden of disease due to VRE bloodstream infections^7^.

The spread of VRE within healthcare settings is attributed to both clonal transmission and horizontal gene transfer (HGT) events^8^. So far, various genotypes of vancomycin resistance have been described, with VanA- and VanB-types being the most relevant^9^. Complementing the known HGT mechanisms of transformation, conjugation, and transduction, bacterial membrane vesicles (BMVs) have been described as vectors for gene transfer in recent years, introducing a fourth pathway, termed vesiduction^10^.

BMVs were first discovered as outer membrane vesicles (OMVs) in Gram-negative bacteria^11^. Distinctly delayed, they were also found in microorganisms with cell walls, such as Gram-positive bacteria^12^, designated as membrane vesicles (MVs). Consequently, MVs still leave many questions unanswered and offer great research potential. BMVs appear to be relevant in a variety of areas, such as communication, immunological processes, biofilm formation, toxicity, and gene transfer^13, 14^, e.g. the transfer of antibiotic resistance genes. The latter has already been validated for OMVs from *Escherichia* *coli*^15–17^, *Klebsiella pneumoniae*^18^, *Acinetobacter baylyi*^19^ and *Acinetobacter* *baumannii*^20, 21^.

Regarding *E. faecium,* the release of MVs has just recently been confirmed^22^. So far, the potential of MVs as a vector for HGT remains hypothetical. Therefore, our study aims to elucidate for the first time whether MVs, released by different VRE, transfer vancomycin resistance to VSE in vitro.

## Results

### Characteristics of VRE isolates and released MVs

Vancomycin resistance of all investigated isolates was confirmed both phenotypically and genotypically (Table 1).

**Table 1:** Phenotypic and genotypic vancomycin resistance of VRE isolates and reference strains.

	MIC vancomycin [µg/ml]	MIC teicoplanin [µg/ml]	*van*-resistance (eazyplex® VRE)
*E. faecalis* **ATCC 29,212**	4	1.5	-
*E. faecium* **ATCC 6057**	0.5	1.5	-
*E. faecium* **ST80**	> 256	1.5	*vanB*
*E. faecium* **ST117**	> 256	32	*vanA*
*E. faecium* **ST192**	> 256	2	*vanB*
*E. faecium* **ST203**	> 256	> 256	*vanA*
*E. faecium* **ST721**	> 256	> 256	*vanA*
*E. faecium* **ST1489**	> 256	2	*vanB*

**Figure 1:**
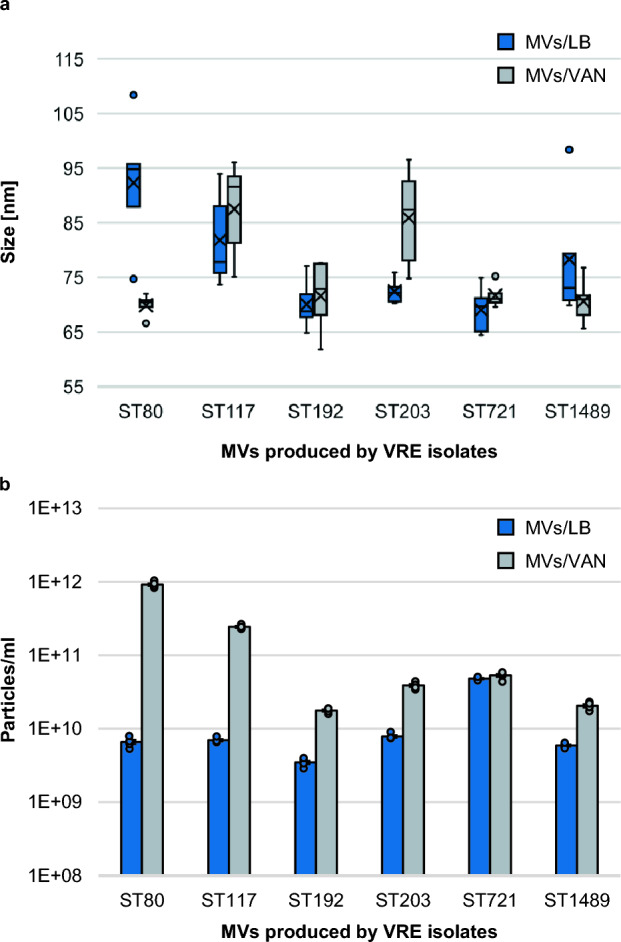
Particle sizes (**a**) and concentrations (**b**) of representative membrane vesicle (MV) samples derived from vancomycin-resistant enterococci (VRE). MVs were isolated from VRE of sequence types (STs) ST80, ST117, ST192, ST203, ST721 and ST1489 defined by multilocus sequence typing cultivated either in lysogeny broth (LB) (MVs/LB) or in LB supplemented with vancomycin (MVs/VAN). Nanoparticle tracking analysis (NTA) measurements consisted of five measurement cycles lasting 60 s each. The mean value of the most frequently measured particle sizes (**a**) is represented by a cross and the median by a line. Whiskers indicate the maximum and minimum values provided they lie within 1.5-fold of the interquartile range, depicted by the box. Dots visualise outliers. The quartile calculation included the median. Particle concentrations (**b**) are given as mean ± standard error. The dots mark the concentrations determined in the individual measuring cycles. For error analysis, the standard evaluation of the NanoSight NTA 3.4 software was used.

**Figure 2:**
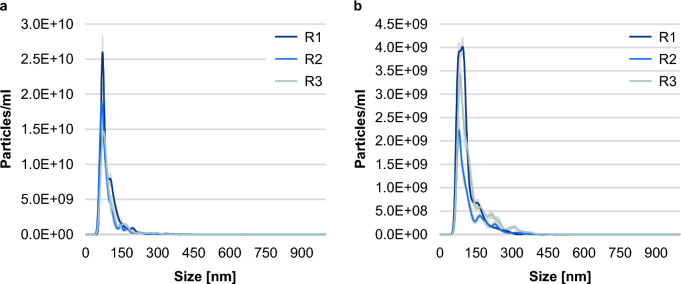
| Exemplary size distribution of representative membrane vesicle (MV) samples derived from vancomycin-resistant enterococci (VRE). MVs were gained under vancomycin stress (MVs/VAN) produced by VRE isolates of sequence types (STs) ST80 (**a**) and ST117 (**b**). In both cases (**a**, **b**), three biological replicates (R1–R3) were examined by nanoparticle tracking analysis (NTA) in five measurement cycles of 60 s each. The results are presented as mean ± standard error, shown as shading. For error analysis, the standard evaluation of the NanoSight NTA 3.4 software was used.

Characterisation of MVs in terms of particle size and concentration via nanoparticle tracking analysis (NTA) revealed differences between MV samples depending on multilocus sequence typing (MLST) sequence types (STs) and cultivation conditions. The most frequently measured particle size ± standard error (SE) ranged from 69.1 ± 2.0 nm for MVs released by ST721 after cultivation in lysogeny broth (LB) (MVs/LB) up to 92.3 ± 5.5 nm (MVs/LB of ST80) (Fig. 1a). Results expanded by the mean particle sizes can be found in Supplementary Figure S1. Size distributions of MVs released by ST80 and ST117 cultured in LB supplemented with vancomycin at subinhibitory concentrations (MVs/VAN) are shown in Fig. 2.

The mean particle concentration ± SE of MV samples varied between 3.48 × 10^9^ ± 1.88 × 10^8^ particles/ml (MVs/LB of ST192) and 9.17 × 10^11^ ± 3.50 × 10^10^ particles/ml (MVs/VAN of ST80) (Fig. 1b). We recorded higher particle concentrations under vancomycin stress for all STs, with the most striking increase for ST80 (139-fold from 6.60 × 10^9^ ± 4.11 × 10^8^ particles/ml for MVs/LB to 9.17 × 10^11^ ± 3.50 × 10^10^ for MVs/VAN) and ST117 (35-fold from 6.99 × 10^9^ ± 2.25 × 10^8^ for MVs/LB to 2.44 × 10^11^ ± 6.76 × 10^9^ for MVs/VAN).

### PCR of MVs

**Figure 3:**
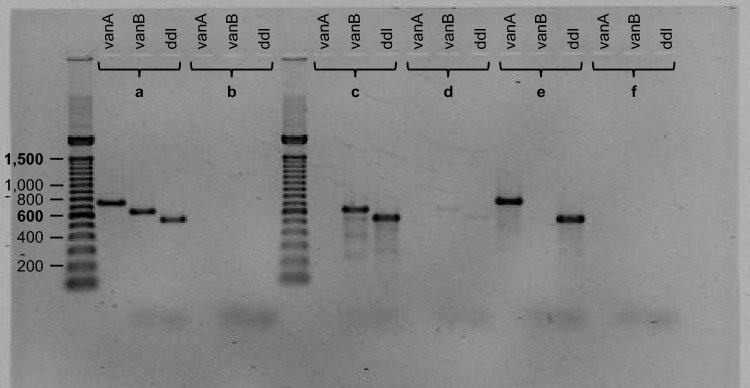
Resistance-encoding DNA fragments of membrane vesicle (MV) samples. MVs were gained under vancomycin stress (MVs/VAN) produced by vancomycin-resistant enterococci (VRE) isolates of sequence types (STs) ST80 and ST117. (**a**) bacterial DNA as a positive control, (**b**) water as a negative control, (**c**) MVs/VAN of isolate ST80, (**d**) MVs/VAN of isolate ST80 post 8 U DNase I treatment, (**e**) MVs/VAN of isolate ST117, and (**f**) MVs/VAN of isolate ST117 post 8 U DNase I treatment. The size of the DNA fragments [bp] is indicated on the left (gel cropped at the lower edge; for full-length gel see Supplementary Figure S2).

PCR of MVs/VAN released by ST80 and ST117, respectively, revealed clearly detectable DNA bands in samples prior to DNase treatment. After removing extravesicular DNA by DNase treatment and subsequent heat lysis of MVs, only weak bands were found consistently in samples MVs/VAN of ST80, while weak bands were only irregularly detectable in samples MVs/VAN of ST117, indicating very low amounts of DNA near or below the detection limit. Figure 3 exemplifies representative samples of MVs/VAN derived from ST80 with weak bands and from ST117 without visible bands.

### Whole genome sequencing (WGS) of MVs

After DNase treatment of MVs/VAN derived from VRE of ST80 and ST117 with subsequent DNA isolation, double-stranded (ds) DNA concentrations amounted to 0.029 ng/µl (ST80) and 0.040 ng/µl (ST117), which was too low for subsequent WGS. Analysis of WGS of DNA isolated from untreated MVs/VAN of VRE isolates ST80 and ST117 showed that for ST80, *vanB* was detected on the chromosome while *vanA* was located on a plasmid in the ST117 isolate and MV DNA. Pairwise alignments of sequences from both MVs and their respective donor isolates showed that the *van* cassettes were complete and identical.

### Exposure of VSE to MVs and bacterial supernatant

For exposure experiments, we determined the mean colony forming units (CFU) ± standard deviation (SD) of *E. faecium* ATCC 6057 under our specific test parameters at 4.65*10^7^ ± 3.29*10^6^ CFU/ml.

After coincubation of both MVs/VAN released from VRE isolates ST80 and ST117 with vancomycin-susceptible *E. faecium* ATCC 6057 at an MV-bacteria ratio of 1,000 and 10,000, no bacterial growth on VRE selective agar was visible indicating no phenotypical detectable *van* resistance inclusion. Controls on Columbia blood agar showed *E. faecium* typical growth.

Similar to the MV experiments, after exposure to supernatants, no growth on VRE selective agar but on Columbia blood agar was observed.

## Discussion

In the hospital setting, VRE pose a threat to particularly vulnerable patient groups, as adequate treatment is only possible using last-resort antibiotics. Due to increasing incidences and resistance, new therapeutic and infection prevention approaches are urgently needed. Our study aimed to assess the risk associated with VRE-derived MVs as a potential vehicle for *van* gene transmission.

We were able to successfully prepare MVs from all investigated VRE isolates. After coincubation of VSE with VRE-derived supernatants and MV samples, no phenotypically effective transfer of vancomycin resistance was observed neither by vesiduction nor by transformation. This is remarkable since at least WGS of DNase-untreated MV samples showed that all *van* resistance-determining genes were present and intact. However, our PCR experiments provide indications that only very small DNA quantities near or below the PCR detection limit were present intravesicularly, hampering vesiduction of vancomycin resistance. Nonetheless, literature has shown that many BMVs contain DNA, albeit conceivably in smaller quantities than extravesicular BMV-associated DNA^23^. Also, the fusion of MVs and vesiductants might be hindered due to species or genotype specificity or cell wall properties that completely differ from those of Gram-negative bacteria. However, fusion of MVs and bacterial cells has already been detected in the Gram-positive *Bacillus subtilis*^24^ and as HGT via MVs in *Ruminococcus* species^25^, assuming resistance transfer is not being prevented by the peptidoglycan layer of Gram-positive bacteria. Investigations regarding the gene transfer ability of BMVs did not inevitably lead to a confirmation^26–28^, suggesting that vesiduction is a complex process which is not yet fully understood. Partly, resistance was selectively transferred^29^, assuming mechanisms for which certain conditions need to be fulfilled^21^.

NTA characterisation of MVs could confirm previous findings, including reported particle sizes^22, 30, 31^. We observed an increased vesicle release under vancomycin stress conditions that has already been witnessed for MVs from *E. faecium*^30^. The reason is suspected to be a weakening of the cell wall caused by vancomycin below inhibitory concentrations^30, 32^. Interestingly, with the help of NTA, we were able to show for the first time that there are intra-species differences regarding the MV release of VRE depending on the ST. VRE of the genotypes ST80 and ST117 release significantly more vesicles under vancomycin stress compared to VRE of other STs (Fig. 1b). These STs may have properties that could provide a selection advantage depending on the enterococcal MV function, hitherto rarely studied. In accordance with this, ST80 and ST117 are currently responsible for the majority of invasive VRE infections in Germany^33^. In several regions, ST117 was reported to be strikingly dominant^34–37^, but no distinct reason, like specific virulence factors^35^ or colonisation persistence^38^, could yet be identified. The increased virulence potential of ST80- and ST117-positive VRE might be due to the elevated MV release itself. MVs have been shown to be crucial modulators of inflammatory responses in a wide range of host tissues^39^ that can substantially contribute to a dysregulated host response. Hence, the enlarged MV quantities might enable ST80- and ST117-positive VRE to contribute to the severity of human infection and inflammation. This would open up new infection prevention options, namely MV-based vaccine development^40^. Since MVs are native antigen-presenting particles harbouring a high immunogenic capacity and lacking replicating potential, they seem to be the perfect target structures for such approaches. These have already been successful in controlling Gram-negative pathogens, e.g. meningococcal B strains^39^.

We would like to address the limitations of our work. First, we used only a single VRE isolate representing each MLST ST. Hence, there might be differences regarding strain and MV characteristics. However, we deliberately chose investigated isolates that are representative of isolates connected to cluster or outbreak events^41^. Second, characterisation using NTA identifies particles that do not necessarily represent vesicles. But, due to distinct peaks in the NTA measurements, it can be assumed that the samples consist predominantly of MVs. Third, as we inconsistently detected weak DNA bands after PCR of DNase-treated MV samples, we cannot rule out artificial measurements, e.g. due to incomplete DNA degradation. Fourth, with the help of the chosen methods, information about vesicle integrity is not provided. Nevertheless, we have no reasonable indication of any integrity disturbance as we followed published protocols and avoided MV treatments redundant for our research^20^.

Summarising, we noticed differences between the MV characteristics depending on the ST of VRE. Especially, enterococci assigned to ST80 and ST117 released markedly more vesicles under vancomycin stress than other isolates investigated. Further research is necessary to uncover ST-specific differences in vesiculation to better understand host–pathogen interactions in terms of invasive VRE infections, their therapy and infection prevention approaches.

## Materials and methods

### Bacterial isolates and growth conditions

Six different VRE isolates defined by MLST (ST80, ST117, ST192, ST203, ST721, and ST1489) were used for MV preparation. The isolates originated from a surveillance sample collection of the University Hospital Münster, Germany, and all of them were derived from routine anal swab samples. Based on previous investigations by Correa-Martinez et al. addressing the spread of VRE in the hospital environment^41^, representative isolates were selected for the respective STs. *E. faecalis* ATCC 29212 and *E. faecium* ATCC 6057 served as vancomycin-susceptible reference strains; *E. faecium* ATCC 6057 was also used as a potential vesiductant.

For general cultivation purposes, all bacteria were grown on Columbia blood agar (Thermo Scientific, Waltham, USA) and incubated at 37 °C for approximately 24 h.

They were further cultured either in LB (Carl Roth, Karlsruhe, Germany) or on LB agar (Carl Roth) to investigate vesiculation under stressful growth conditions^22^.

### Determination of phenotypical and genotypical resistance of bacterial isolates

Phenotypic resistance was identified by detecting the minimum inhibitory concentrations (MICs). Vancomycin and teicoplanin test strips (Liofilchem, Roseto degli Abruzzi, Italy) were placed on Mueller–Hinton agar (Thermo Scientific) to which a bacterial suspension in 0.9% NaCl (B. Braun, Melsungen, Germany) with a McFarland value of 0.5, measured by using the Densimat (Biomérieux, Marcy-l’Étoile, France) densitometer, was applied. After incubation at 35 °C for 24 h, MICs were compared to the clinical breakpoints given by the European Committee on Antimicrobial Susceptibility Testing at its latest version^42^. The genotypic resistance of every isolate was confirmed using the eazyplex® VRE platform (AmplexDiagnostics, Gars Bahnhof, Germany).

### MV preparation

MV preparation from VRE isolates was carried out as previously described with slight modifications^16^. Bacterial isolates were grown in 500 ml LB at 37 °C with shaking at 180 rpm until stationary phase (16 h). After centrifugation (5,525 g, 4 °C, 20 min) in the Allegra™ 25R centrifuge with a TS-5.1–500 rotor (Beckman Coulter, Brea, USA), supernatants were passed through a 0.22 µm sterile filter (Merck, Darmstadt, Germany). MVs were pelleted by ultracentrifugation (235,000 g, 4 °C, 2 h) using the Optima™ XPN-80 ultracentrifuge with a Type 45 Ti rotor (Beckman Coulter) and subsequently resuspended in 20 mM TRIS–HCl buffer (pH 8.0). For additional preparation of MVs under vancomycin stress (MVs/VAN), LB of the main culture was supplemented with 30 µg/ml vancomycin (Demo Pharmaceuticals, Hallbergmoos, Germany and Hikma Pharmaceuticals, London, UK), which is in the range of therapeutic serum concentrations^43^. Sterility was tested by spreading 10 µl of sample on Columbia blood agar and incubating it at 37 °C for at least 24 h. MVs were stored at 4 °C until further use.

### Characterisation of MVs via NTA

MVs were further characterised by using the NanoSight NS300 (Malvern Panalytical, Malvern, UK) with a 488 nm laser for NTA to determine particle size and concentration. Prior to application of NTA, the samples were diluted in phosphate buffered saline (PBS) (pH 7.4) (Sigma-Aldrich, St. Louis, USA). Measurements were performed according to the manufacturer’s recommendations and consisted of five runs lasting 60 s each at the following settings: camera level 16, temperature 25 °C, syringe pump speed 30 and detection threshold 5. Data were analysed using version 3.4.4 of NanoSight NTA 3.4 software (Malvern Panalytical). When reporting results, the program's standard evaluation was used. Exemplifying our overall experiments, we characterised three biological replicates of MVs/VAN derived from ST80 and ST117 (Fig. 2).

### PCR of MVs

A standard PCR was used to determine whether MVs/VAN of ST80 and ST117 contained DNA fragments of *vanA*, *vanB* or *ddl*. *Ddl*, as a species-specific gene for *E. faecium*, served as a control. Samples were additionally treated with DNase to degrade extravesicular DNA. Two different test conditions were conducted, each in 100 µl total reaction volume. Vesicles were incubated with 4 U DNase I (New England Biolabs, Ipswich, USA) for 10 min at 37 °C and separately with 8 U DNase I for 30 min at 37 °C. DNase I was inactivated at 75 °C for 10 min. Samples with initial extravesicular dsDNA concentrations over 45 ng/µl were diluted in 20 mM TRIS–HCl buffer (pH 8.0) prior to DNase treatment. For this purpose, dsDNA concentration was determined using the Qubit™ 1X dsDNA High Sensitivity (HS) Assay Kit (Invitrogen, Waltham, USA) following the application instructions. Relative fluorescence units were measured by the DS-11 FX + Spectrophotometer/Fluorometer (DeNovix, Wilmington, USA).

MVs were heat-lysed at 95 °C for 10 min prior to PCR. For each reaction, the PCR master mix consisted of 10.17 µl Milli-Q® water (Merck), 12.5 µl REDTaq® ReadyMix™ (Sigma-Aldrich), and 0.67 µl of forward and reverse primers (Sigma-Aldrich) each (initial concentration of 30 µM). Primers (Table 2) were either used from the literature (*vanA*^44^, *ddl*^45^) or designed using Primer-BLAST^46^ (*vanB*; accession number AF550667.1). 2 µl of sample in a total reaction volume of 25 µl underwent the following PCR protocol in the T100™ Thermal Cycler (Bio-Rad, Hercules, USA): initial denaturation at 94 °C for 3 min, 30 PCR cycles (denaturation at 94 °C for 1 min, primer annealing at 54 °C for 1 min and elongation at 72 °C for 1 min), and final elongation at 72 °C for 7 min^47^. Milli-Q® water served as a negative control, while bacterial DNA of VRE isolates functioned as a positive control. For bacterial DNA extraction, a single VRE colony was suspended in 5% Chelex® 100 (Bio-Rad) and incubated at 100 °C for 10 min. After centrifugation (13,400 g, 19 °C, 3 min) using the Centrifuge 5418 R with the FA-45-18-11 rotor (Eppendorf, Hamburg, Germany), the supernatant was removed and used as PCR template. PCR reaction products were subsequently transferred to a 1.5% agarose gel (Carl Roth) and separated at 80 V for 2 h, according to PCR product length, in 0.5 TBE running buffer (Merck) in the DNA Sub Cell (Bio-Rad). A 100 bp DNA ladder (Invitrogen) was used as size marker. The gels were stained in 0.5 µg/ml concentrated ethidium bromide solution (Bio-Rad) and analysed with the Gel Doc™ 2000 (Bio-Rad).

**Table 2:** Oligonucleotide primers.

Gene	Primer pair ^a^	Sequence (5'–3')	PCR product length [bp]	Source
*vanA*	vanA_1_	GGGAAAACGACAATTGC	732	^44^
	vanA_2_	GTACAATGCGGCCGTTA		
*vanB*	vanB_1_	TTCGATCCGCACTACATCGG	648	This study
	vanB_2_	GCCTTTTTCCGGCTCGTTTT		
*ddl*	ddl_1_	GAGACATTGAATATGCCTTATG	560	^45^
	ddl_2_	AAAAAGAAATCGCACCG		

^a^_1_Forward primer; _2_Reverse primer.

### WGS of VRE isolates and MVs

WGS of the donor isolates was performed on the MiSeq platform (Illumina, San Diego, USA) using a Nextera XT library according to the manufacturer’s recommendations. The resulting reads were de novo assembled using Velvet v1.1.04 and trimmed to an average quality of 30 in a window of 20.

DNA was isolated from MVs/VAN of VRE isolates ST80 and ST117, both untreated and post 4 U DNase I treatment, using the Monarch® Genomic DNA Purification Kit (New England Biolabs). The protocol for genomic DNA purification from Gram-positive bacteria was applied according to the manufacturer’s instructions, omitting the lysozyme treatment. For WGS, samples were adjusted to dsDNA concentrations above 20 ng/µl and not sequenced if below.

WGS of the DNA isolated from the untreated MVs/VAN of ST80 and ST117 was performed using the Pacific Biosciences Sequel Ile platform (Pacific Biosciences, Menlo Park, USA). For sequencing on the platform, a library was created with the SMRTbell Express Template Prep Kit 2.0 (Pacific Biosciences) according to the manufacturer’s recommendations. For de novo assembly, the resulting HiFi long-reads were prepared using the microbial assembly pipeline within the SMRT Link software version 11 (Pacific Biosciences).

The assembled contigs of both VRE isolates and MVs were screened for the presence of the *vanA* and *vanB* cassettes using the AMRFinderPlus^48^ implementation in Ridom SeqSphere+^49^. The contigs containing the *van* cassettes were extracted from the assemblies of MVs and annotated using Bakta v1.7.0^50^. Subsequently, the annotated contigs were pairwise aligned with the assemblies of their respective donor isolates using Mauve v2.4.0^51^. The resulting alignments were manually examined for sequence identity and completeness of the *van* cassettes.

### CFU determination of VSE

To estimate the number of living bacteria of the potential vesiductant *E. faecium* ATCC 6057 under our specific test parameters, an overnight grown culture (16 h, 37 °C, 180 rpm) was adjusted to an OD_600_ of 0.1. OD_600_ was ascertained by the DS-11 FX + Spectrophotometer/Fluorometer (DeNovix). After serial dilution in PBS, 50 µl were plated on Columbia blood agar by using glass beads. After 24 h of incubation at 37 °C, CFU/ml was calculated as the mean ± SD of three dilution levels. The experiment was repeated three times.

### Preparation of sterile bacterial supernatants

Liquid cultures of VRE isolates ST80 and ST117 were grown overnight (16 h) in LB at 37 °C with shaking at 180 rpm. Bacteria were removed by centrifugation (5,525 g, 4 °C, 5 min) in the Allegra™ 25R centrifuge with a TS-5.1–500 rotor (Beckman Coulter), supernatants were collected and sterile filtered using a 0.2 µm syringe filter (Corning, Corning, USA). A sterile control was set up on LB agar.

### Exposure of VSE to MVs and bacterial supernatant

Exposure experiments were performed with MVs/VAN and supernatants of VRE isolates ST80 and ST117 adapted from previously published methods^16, 21^. MVs were used for exposure within the first eight days of storage, whereas the supernatants were freshly prepared prior to the experiment. Initially, a single colony of strain *E. faecium* ATCC 6057 was cultivated overnight (16 h) in LB at 37 °C with shaking at 180 rpm. The OD_600_ of the bacterial culture was adjusted to 0.1 by dilution in LB broth. Exposure of VSE to MVs was performed at two concentrations: 1,000 and 10,000 particles per bacterium respectively. 500 µl LB, 100 µl bacterial culture and MVs at the respective concentrations were combined. The mixture was replenished with 20 mM TRIS–HCl buffer (pH 8.0) to a total volume of 1 ml.

For exposure to supernatants, 100 µl adjusted bacterial culture was combined with 900 µl supernatant. After incubation at 37 °C for 4 h without shaking, followed by 4 h with shaking at 180 rpm, 24 ml of fresh LB was added to the culture. After overnight cultivation (16 h) at 37 °C with shaking at 180 rpm, bacteria were pelleted by centrifugation (1,500 g, 25 °C, 2 min) in the Allegra™ 25R centrifuge with a TS-5.1–500 rotor (Beckman Coulter) and resuspended in 1 ml LB. 100 µl each were plated five times on VRE selective agar (Bio-Rad) by using glass beads. The bacteria were further serially diluted in PBS and spread on Columbia blood agar. Each experiment was repeated twice.

## Ethical approval

Human participants were not involved in the present study. A statement of the ethical committee approving the experiments was therefore not included.

### Supplementary Information

Supplementary Information.

## Data Availability

Whole genome sequencing data of analysed VRE genomes were submitted to NCBI database (BioProject ID: PRJNA980408; https://www.ncbi.nlm.nih.gov/bioproject/PRJNA980408).

## References

[1] O'Neill, J. Tackling drug-resistant infections globally: final report and recommendations. https://apo.org.au/sites/default/files/resource-files/2016-05/apo-nid63983.pdf (2016).

[2] World Health Organization. Antimicrobial resistance: global report on surveillance. https://apps.who.int/iris/bitstream/handle/10665/112642/9789241564748_eng.pdf;jsessionid=A52206751F0E10B7E93A364D474DCF99?sequence=1 (2014).

[3] Tacconelli E (2018). Discovery, research, and development of new antibiotics: the WHO priority list of antibiotic-resistant bacteria and tuberculosis. Lancet Infect. Dis..

[4] García-Solache M, Rice LB (2019). The enterococcus: a model of adaptability to its environment. Clin. Microbiol. Rev..

[5] Prematunge C (2016). VRE and VSE bacteremia outcomes in the era of effective VRE therapy: a systematic review and meta-analysis. Infect. Control Hosp. Epidemiol..

[6] European Centre for Disease Prevention and Control. Antimicrobial resistance in the EU/EEA (EARS-Net)—Annual Epidemiological Report 2021. https://www.ecdc.europa.eu/sites/default/files/documents/AER-EARS-Net-2021_2022-final.pdf (2022).

[7] Brinkwirth S (2022). Germany’s burden of disease of bloodstream infections due to vancomycin-resistant *Enterococcus faecium* between 2015–2020. Microorganisms.

[8] Arredondo-Alonso S, Top J, Corander J, Willems RJL, Schürch AC (2021). Mode and dynamics of *vanA*-type vancomycin resistance dissemination in Dutch hospitals. Genome Med..

[9] Stogios PJ, Savchenko A (2020). Molecular mechanisms of vancomycin resistance. Protein Sci..

[10] Soler N, Forterre P (2020). Vesiduction: the fourth way of HGT. Environ. Microbiol..

[11] Knox KW, Vesk M, Work E (1966). Relation between excreted lipopolysaccharide complexes and surface structures of a lysine-limited culture of *Escherichia coli*. J. Bacteriol..

[12] Lee E-Y (2009). Gram-positive bacteria produce membrane vesicles: Proteomics-based characterization of *Staphylococcus aureus*-derived membrane vesicles. Proteomics.

[13] Jan AT (2017). Outer membrane vesicles (OMVs) of gram-negative bacteria: a perspective update. Front. Microbiol..

[14] Cao Y, Lin H (2021). Characterization and function of membrane vesicles in Gram-positive bacteria. Appl. Microbiol. Biotechnol..

[15] Yaron S, Kolling GL, Simon L, Matthews KR (2000). Vesicle-mediated transfer of virulence genes from *Escherichia coli* O157:H7 to other enteric bacteria. Appl. Environ. Microbiol..

[16] Bielaszewska M, Daniel O, Karch H, Mellmann A (2020). Dissemination of the *bla*_*CTX-M-15*_ gene among Enterobacteriaceae via outer membrane vesicles. J. Antimicrob. Chemother..

[17] Li C (2022). Outer membrane vesicles of *Avian Pathogenic Escherichia coli* mediate the horizontal transmission of *bla*_*CTX-M-55*_. Pathogens.

[18] Dell'Annunziata F (2021). Outer membrane vesicles derived from *Klebsiella pneumoniae* are a driving force for horizontal gene transfer. Int. J. Mol. Sci..

[19] Fulsundar S (2014). Gene transfer potential of outer membrane vesicles of *Acinetobacter baylyi* and effects of stress on vesiculation. Appl. Environ. Microbiol..

[20] Rumbo C (2011). Horizontal transfer of the OXA-24 carbapenemase gene via outer membrane vesicles: A new mechanism of dissemination of carbapenem resistance genes in *Acinetobacter baumannii*. Antimicrob. Agents Chemother..

[21] Chatterjee S, Mondal A, Mitra S, Basu S (2017). *Acinetobacter baumannii* transfers the *bla*_*NDM-1*_ gene via outer membrane vesicles. J. Antimicrob. Chemother..

[22] Wagner T (2018). *Enterococcus faecium* produces membrane vesicles containing virulence factors and antimicrobial resistance related proteins. J. Proteomics.

[23] Bitto NJ (2017). Bacterial membrane vesicles transport their DNA cargo into host cells. Sci. Rep..

[24] Kim Y, Edwards N, Fenselau C (2016). Extracellular vesicle proteomes reflect developmental phases of *Bacillus subtilis*. Clin. Proteomics.

[25] Klieve AV (2005). Naturally occurring DNA transfer system associated with membrane vesicles in cellulolytic *Ruminococcus *spp. of ruminal origin. Appl. Environ. Microbiol..

[26] Renelli M, Matias V, Lo RY, Beveridge TJ (2004). DNA-containing membrane vesicles of *Pseudomonas aeruginosa* PAO1 and their genetic transformation potential. Microbiology (Reading).

[27] González LJ (2016). Membrane anchoring stabilizes and favors secretion of New Delhi metallo-β-lactamase. Nat. Chem. Biol..

[28] Kim SW (2018). Outer membrane vesicles from β-lactam-resistant *Escherichia coli* enable the survival of β-lactam-susceptible *E*. *coli* in the presence of β-lactam antibiotics. Sci. Rep..

[29] Lee AR (2022). Membrane vesicles from antibiotic-resistant *Staphylococcus aureus* transfer antibiotic-resistance to antibiotic-susceptible *Escherichia coli*. J. Appl. Microbiol..

[30] Kim MH (2019). Production of membrane vesicles by *Enterococcus faecium* cultured with or without subinhibitory concentrations of antibiotics and their pathological effects on epithelial cells. Front. Cell. Infect. Microbiol..

[31] Costantini PE (2021). Extracellular vesicles generated by gram-positive bacteria protect human tissues *Ex Vivo* From HIV-1 infection. Front. Cell. Infect. Microbiol..

[32] Liu X (2022). Research progress on bacterial membrane vesicles and antibiotic resistance. Int. J. Mol. Sci..

[33] Robert Koch Institut. Epidemiologisches Bulletin 27/2021. https://www.rki.de/DE/Content/Infekt/EpidBull/Archiv/2021/Ausgaben/27_21.pdf?__blob=publicationFile (2021).

[34] Falgenhauer L (2019). Near-ubiquitous presence of a vancomycin-resistant *Enterococcus faecium* ST117/CT71/*vanB* –clone in the Rhine-Main metropolitan area of Germany. Antimicrob. Resist. Infect. Control.

[35] Weber A, Maechler F, Schwab F, Gastmeier P, Kola A (2020). Increase of vancomycin-resistant *Enterococcus faecium* strain type ST117 CT71 at Charité–Universitätsmedizin Berlin, 2008 to 2018. Antimicrob. Resist. Infect. Control.

[36] Werner G (2020). Thirty years of VRE in Germany—“expect the unexpected”: The view from the national reference centre for staphylococci and enterococci. Drug Resist. Updat..

[37] Falgenhauer L (2021). Changing epidemiology of vancomycin-resistant *Enterococcus faecium*: Results of a genome-based study at a regional neurological acute hospital with intensive care and early rehabilitation treatment. Infect. Prev. Pract..

[38] Correa-Martinez CL (2019). Risk factors for long-term vancomycin-resistant enterococci persistence—A prospective longitudinal study. Microorganisms.

[39] Zingl FG, Leitner DR, Thapa HB, Schild S (2021). Outer membrane vesicles as versatile tools for therapeutic approaches. Microlife.

[40] Wagner TM (2023). Enterococcal membrane vesicles as vaccine candidates. Int. J. Mol. Sci..

[41] Correa-Martinez CL, Tönnies H, Froböse NJ, Mellmann A, Kampmeier S (2020). Transmission of vancomycin-resistant enterococci in the hospital setting: Uncovering the patient-environment interplay. Microorganisms.

[42] The European Committee on Antimicrobial Susceptibility Testing. Breakpoint tables for interpretation of MICs and zone diameters. Version 13.0. https://www.eucast.org/fileadmin/src/media/PDFs/EUCAST_files/Breakpoint_tables/v_13.0_Breakpoint_Tables.pdf (2023).

[43] Rybak MJ (2006). The pharmacokinetic and pharmacodynamic properties of vancomycin. Clin. Infect. Dis..

[44] Dutka-Malen S, Evers S, Courvalin P (1995). Detection of glycopeptide resistance genotypes and identification to the species level of clinically relevant enterococci by PCR. J. Clin. Microbiol..

[45] Homan WL (2002). Multilocus sequence typing scheme for *Enterococcus faecium*. J. Clin. Microbiol..

[46] Ye J (2012). Primer-BLAST: a tool to design target-specific primers for polymerase chain reaction. BMC Bioinform..

[47] Depardieu F, Perichon B, Courvalin P (2004). Detection of the *van* alphabet and identification of enterococci and staphylococci at the species level by multiplex PCR. J. Clin. Microbiol..

[48] Feldgarden M (2021). AMRFinderPlus and the Reference Gene Catalog facilitate examination of the genomic links among antimicrobial resistance, stress response, and virulence. Sci. Rep..

[49] Jünemann S (2013). Updating benchtop sequencing performance comparison. Nat. Biotechnol..

[50] Schwengers O (2021). Bakta: rapid and standardized annotation of bacterial genomes via alignment-free sequence identification. Microb. Genom..

[51] Darling ACE, Mau B, Blattner FR, Perna NT (2004). Mauve: multiple alignment of conserved genomic sequence with rearrangements. Genome Res..

