# Supplementary material for: Role of membrane vesicles in the transmission of vancomycin resistance in Enterococcus faecium
Source: Sci Rep. 2024 Jan 22;14:1895. doi: 10.1038/s41598-024-52310-1 (PMC10803344; doi:10.1038/s41598-024-52310-1)
Supplement: Supplementary file 1 — Supplementary Information. [file 41598_2024_52310_MOESM1_ESM.pdf]

Role of membrane vesicles in the transmission of vancomycin resistance in *Enterococcus faecium*

Johanna Lehmkuhl[1], Julia Sophie Schneider[1], Kari Lavinia vom Werth[1], Natalie Scherff[1], Alexander Mellmann[1] and Stefanie Kampmeier[1,2]*

[1] Institute of Hygiene, University Hospital Münster, 48149 Münster, Germany

[2] Institute for Hygiene and Microbiology, University of Würzburg, 97080 Würzburg, Germany

* Correspondence: Stefanie.Kampmeier@uni-wuerzburg.de; Phone: +49 931 31 87794

Supplementary information

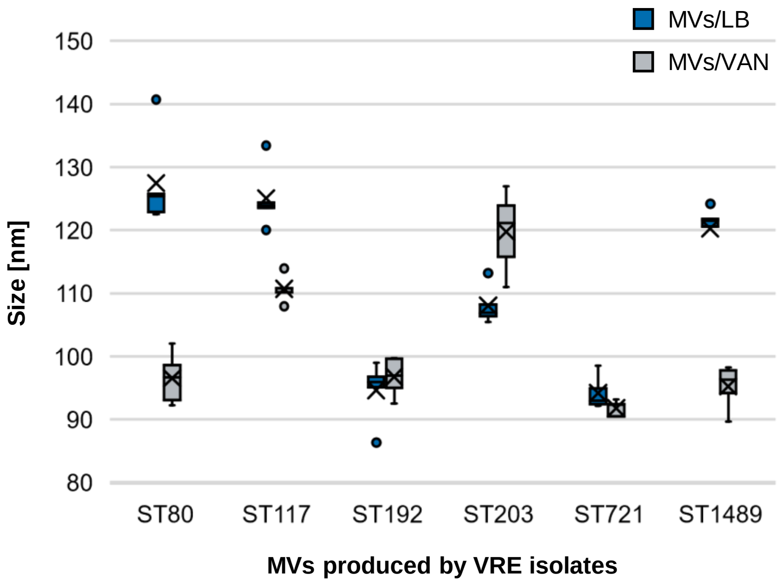

Supplementary Figure S1 | Mean particle sizes of representative membrane vesicle (MV) samples derived by different vancomycin-resistant enterococci (VRE).

MVs were isolated from VRE of sequence types (STs) ST80, ST117, ST192, ST203, ST721 and ST1489 defined by multilocus sequence typing cultivated either in lysogeny broth (LB) (MVs/LB) or in LB supplemented with vancomycin (MVs/VAN).

Nanoparticle tracking analysis (NTA) measurements consisted of five measurement cycles lasting 60 s each.

The mean value of average particle sizes is represented by a cross and the median by a line. Whiskers indicate the maximum and minimum values provided they lie within 1.5-fold of the interquartile range, depicted by the box. Dots visualise outliers. The quartile calculation included the median.

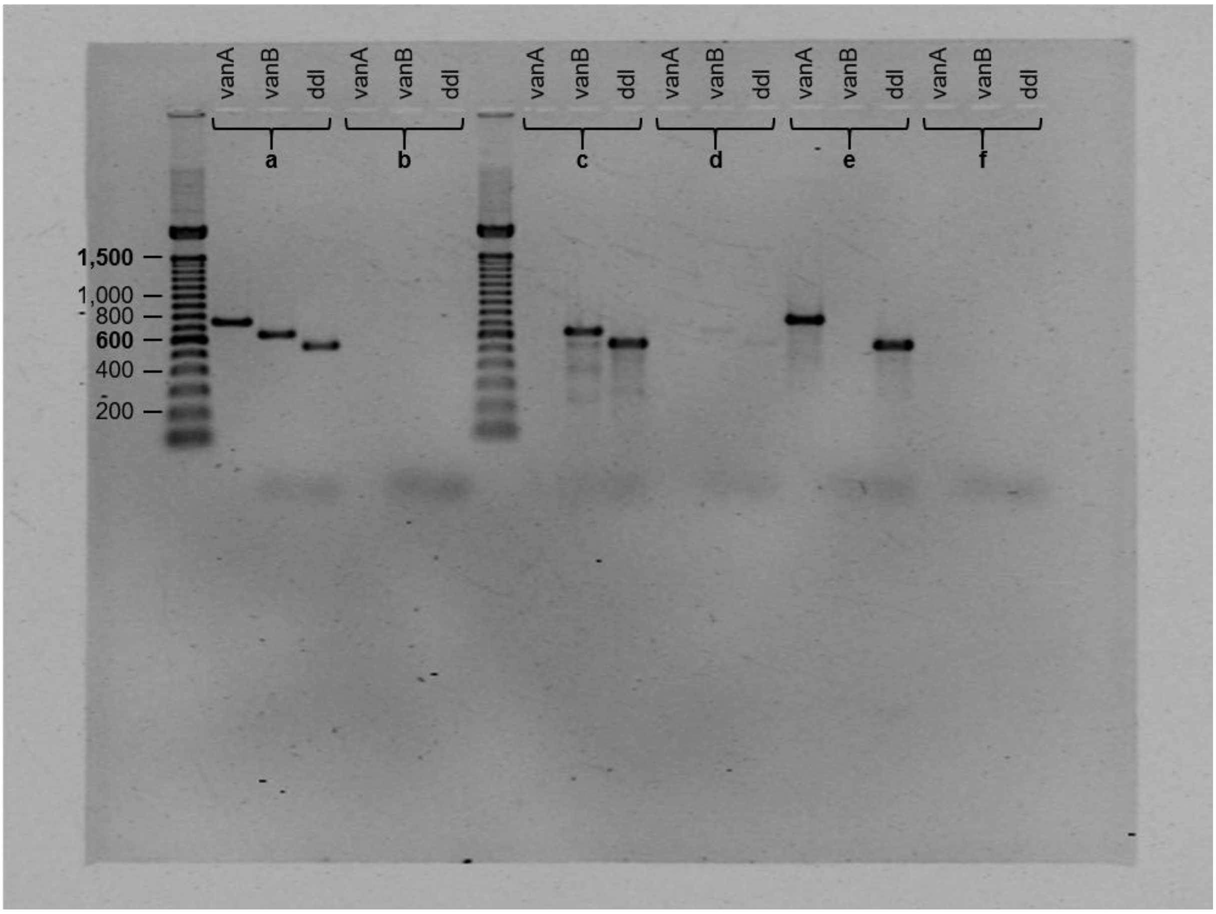

Supplementary Figure S2 | Resistance-encoding DNA fragments of membrane vesicle (MV) samples.

MVs were gained under vancomycin stress (MVs/VAN) produced by vancomycin-resistant enterococci (VRE) isolates of sequence types (STs) ST80 and ST117.

(a) bacterial DNA as a positive control, (b) water as a negative control, (c) MVs/VAN of isolate ST80, (d) MVs/VAN of isolate ST80 post 8 U DNase I treatment, (e) MVs/VAN of isolate ST117, and (f) MVs/VAN of isolate ST117 post 8 U DNase I treatment. The size of the DNA fragments [bp] is indicated on the left (full-length gel; cropped gel is displayed in Figure 3).
